# Comparative study of adenosine 3′‐pyrophosphokinase domains of MuF polymorphic toxins

**DOI:** 10.1002/2211-5463.70038

**Published:** 2025-04-15

**Authors:** Eloïse M. Paulet, Julia Bartoli, Atakan Kabtan, Chloé M. Piras, Audrey C. Tempier, Eric Cascales, Julie P. Viala

**Affiliations:** ^1^ Aix Marseille Univ, CNRS, LISM (UMR7255), IMM Marseille France

**Keywords:** adenosine 3′‐pyrophosphohydrolase, adenosine 3′‐pyrophosphokinase, Apk2, MuF, (p)ppApp, (p)ppGpp

## Abstract

Polymorphic toxins (PT) are multidomain proteins used for interbacterial competition and pathogenesis. The N‐terminal domain of PT specifies the mode of transport and names the family, while the variable C‐terminal domain carries the toxic activity, which can be counteracted by immunity proteins that protect the PT‐producing bacterium. The MuF family of polymorphic toxins is specifically associated with temperate phages, and our recent work showed that the C‐terminal domain of a MuF toxin encoded by a *Streptococcus pneumoniae* prophage carries adenosine 3′‐pyrophosphokinase activity. This type of toxin, which combines a MuF N‐terminal domain and an adenosine 3′‐pyrophosphokinase C‐terminal domain, is called Apk2 for adenosine 3′‐pyrophosphokinase family 2. Here, we extend the characterization of this novel family of toxins by providing information on two new members encoded by prophages of *Mannheimia haemolytica* and *Pasteurella multocida*. Production of their adenosine 3′‐pyrophosphokinase domains (Apk2_tox_) in the heterologous host *Escherichia coli* revealed different levels of toxicity, essentially due to their stability. *In vitro* assays with the purified *M. haemolytica* Apk2_tox_ domain demonstrated that, identically to that of *S. pneumoniae*, it exclusively produces (p)ppApp from ATP. The role of immunity proteins and their interchangeability in cross‐protection and protein–protein interaction assays was tested. While the immunity proteins that hydrolyse pppApp to ATP are interchangeable, those that inhibit the toxin by protein–protein interaction are mainly active against their intrastrain partner. Overall, this study highlights the conserved features of these enzymatic domains, such as their toxicity, their specific activity toward ATP, and their universal and specific immunities.

## Graphical abstract

With the ultimate goal of understanding the association of toxin‐immunity modules to temperate phages, we characterized toxins from three prophages and examined cross‐protection from immunity proteins. The toxins exhibit adenosine 3′‐pyrophosphokinase activity and are toxic in *Escherichia coli*. Immunity proteins that interfere via protein association are mainly orthologous to their cognate partners, while those hydrolyzing the reaction product are universal.

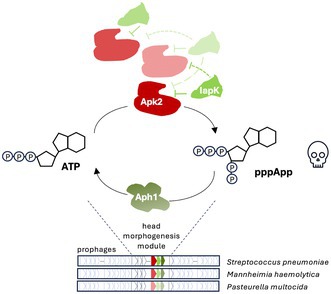

**Abbreviations**
AHTC: anhydrotetracycline
Aph1: adenosine 3′‐pyrophosphohydrolase
Apk2_tox_
: toxic domain of Apk2
Apk(x): adenosine 3′‐pyrophosphokinase family (x)
HD: hydrolysis motif
IapK: immunity of adenosine 3′‐pyrophosphokinase
IPTG: isopropyl β‐d‐thiogalactopyranoside
mhao: *Mannheimia haemolytica*
pmv: *Pasteurella multocida*
PT: polymorphic toxin
RBS: ribosome binding site
RSH: RelA/SpoT homolog
SAH: small alarmone hydrolase
SAS: small alarmone synthetase
SAX‐HPLC: strong anion exchange high‐performance liquid chromatography
snu: *Streptococcus pneumoniae*
syn: synthesis motif
TxSS: type x secretion system

Much studied in the 1970s and then somehow neglected, bacteriophages have been the subject of renewed interest in recent decades. In the context of phagotherapy, lytic phages, which infect and kill bacteria, are considered a possible alternative to the use of antibiotics [1]. By contrast, temperate phages, which integrate their genetic material into the bacterial chromosome of the host, offer an angle of study for understanding genome evolution [2, 3].

Phage genomes usually encode structural components of the phage and proteins and enzymes that subvert the host functions [4, 5]. Recent analyses further showed that phage genomes can encode putative toxins. Indeed, a family of polymorphic toxins (PT) specifically associated with temperate phages has been identified and has a conserved MuF domain on the N‐terminal side [6, 7]. Polymorphic toxins are multidomain proteins involved in pathogenesis and interbacterial competition. They include colicins and subfamilies of type V (T5SS), type VI (T6SS) and type VII (T7SS) secretion system effectors [8]. PT are characterized by a conserved N‐terminal domain involved in toxin transport. Each N‐terminal domain defines a family of PT. These toxins also possess a variable C‐terminal domain that carries the toxic activity. Thus, PT of the MuF family have an N‐terminal conserved MuF domain, named after the GpF protein of the Mu phage, and a C‐terminal extension carrying a predicted toxic activity. *muf* genes are systematically associated with the genes encoding the phage head. Little is known about the MuF proteins except that they are thought to be present in 2–3 copies in the virion head and that they bind viral DNA [9, 10].

Recently, we characterized a member of the MuF PT family encoded by a prophage of the Gram‐positive bacterium *Streptococcus pneumoniae*. We showed that its C‐terminal extension has (p)ppApp synthetase activity and confers toxicity when produced in the *Escherichia coli* heterologous host [11]. This family of proteins that associates an N‐terminal MuF domain with a C‐terminal (p)ppApp synthetase domain is known as Apk2 (adenosine 3′‐pyrophosphokinase family 2) [12]. Another family, called Apk1 (adenosine 3′‐pyrophosphokinase family 1), was also demonstrated to have (p)ppApp synthetase activity, but is associated with a N‐terminal PAAR domain, which is part of the T6SS tip complex [12, 13]. ppApp and pppApp, collectively written (p)ppApp, are modified nucleotides resulting from the addition of a pyrophosphate group from an ATP molecule to the 3′‐hydroxyl end of an ADP and ATP molecule, respectively. Except for the adenosine group instead of the guanosine, (p)ppApp is similar to the (p)ppGpp alarmone, which is synthesized during the stringent response to nutritional stress, such as amino acid deprivation. The accumulation of (p)ppGpp downregulates macromolecule synthesis pathways and upregulates stress control pathways [14, 15]. The production and degradation of (p)ppGpp are regulated by three types of enzymes: RSH (RelA/SpoT homolog), SAS (small alarmone synthetase), and SAH (small alarmone hydrolase) [14, 15]. Like SpoT, well‐characterized in *E. coli*, RSHs possess both catalytic sites associated with (p)ppGpp synthesis and hydrolysis. However, both sites are not always functional: RelA in *E. coli* lacks hydrolase activity due to the substitution of conserved amino acids. SAS and SAH, on the other hand, contain a single catalytic domain, responsible for synthesis and hydrolysis, respectively. The synthesis domain contains five syn (synthesis) motifs involved in the coordination of the magnesium cofactor, ATP, and GTP/GDP. The hydrolysis domain contains six HD (hydrolysis) motifs, involved in the coordination of the manganese cofactor, the guanine base, and the hydrolysis of (p)ppGpp [14, 16]. The (p)ppApp synthetase domain from Apk2 displays Syn motifs similar to those of the (p)ppGpp synthetases, with some sequence variations [11].

In the *S. pneumoniae* prophage genome, the *apk2* gene is followed by the *iapK* and *aph1* genes, which encode immunity proteins. The IapK (immunity of adenosine 3′‐pyrophosphokinase) protein inhibits Apk2 toxin activity by occluding the pyrophosphate acceptor nucleotide‐binding site [11, 13, 17]. Aph1 (adenosine 3′‐pyrophosphohydrolase) hydrolyzes (p)ppApp in a manner similar to (p)ppGpp hydrolases, partially regenerating the cellular pools of ATP and ADP [11, 12]. Here, we extend the characterization of adenosine 3′‐pyrophosphokinases by providing information on two new members of the Apk2 family encoded by the prophages of the Gram‐negative bacteria *Mannheimia haemolytica* and *Pasteurella multocida*. Similar to that of *S. pneumoniae*, their C‐terminal Apk2_tox_ domains conferred toxicity when produced in the heterologous host *E. coli*. The *M. haemolytica* Apk2_tox_ domain was further shown to have (p)ppApp synthetase activity *in vitro*. We then unsuccessfully tried to convert Apk2_tox_ to a (p)ppGpp synthetase through substitution of the Syn motifs. Finally, the interchangeability of the immunity proteins was also investigated by cross‐neutralization and protein–protein interaction assays.

## Materials and methods

### Bacterial strains and media

*Escherichia coli* strains used in this study are listed in Table S1. Bacteria were grown in 2YT, Lysogeny broth (LB) or MacConkey agar (BD, Sparks, MD, USA), in the presence of antibiotics to maintain plasmids (ampicillin 100 μg·mL^−1^, kanamycin 50 μg·mL^−1^ or chloramphenicol 50 μg·mL^−1^).

### Plasmid construction and site‐directed mutagenesis

Plasmids and primers used in this study are listed in Tables S2 and S3, respectively. PCR amplifications were performed with Phusion High‐Fidelity DNA Polymerase (NEB, Evry, France). Site‐directed mutagenesis was performed on plasmids following the instructions of the QuickChange site‐directed mutagenesis kit (Stratagene). DNA templates corresponding to a portion of *S. pneumoniae* SPNA45, *M. haemolytica* D174, and *P. multocida* subsp. *multocida* HN06 prophage genomic regions were sequence‐optimized for *E. coli* and synthesized by IDT. All constructs were checked by DNA sequencing (Eurofins Genomics, Ebersberg, Germany). The sequences of the proteins can be found in the Kyoto Encyclopedia of Genes and Genomes database (https://www.genome.jp/kegg/) using the following organism code and locus tags for *apk2*, *iapK*, and *aph1*, respectively:

snu, SPNA45_00317, SPNA45_00318, and SPNA45_00319 for *S. pneumoniae*;
mhao, J451_00890, J451_00895 and J451_00900 for *M. haemolytica*; and
pmv, PMCN06_2090, PMCN06_2091 and PMCN06_2092 for *P. multocida*.

### Toxicity, survival, and toxicity neutralization assays

These assays were performed as previously described [11]. Toxicity assays: *E. coli* MG1655 cells were transformed with plasmids allowing production of the toxin domain under the control of a P_BAD_ promoter, which is glucose‐locked and arabinose‐induced. For toxicity assays, serial dilutions of bacterial cultures were spotted on LB agar plates containing either 1% glucose or 0.2% arabinose, and the plates were incubated at 37 °C for 18 h. Survival assay: 0.2% arabinose was added to the culture medium while the bacteria were in the exponential phase. Then, at different time points, aliquots were harvested, washed, and serial dilutions were spotted on LB agar plates containing 1% glucose. Toxicity neutralization assays: *E. coli* MG1655 cells were cotransformed with two plasmids, one allowing the production of the toxin domain under the control of a P_BAD_ promoter and the other allowing the production of the immunity protein under the control of an anhydrotetracycline (AHTC)‐inducible P_TET_ promoter. Serial dilutions of bacterial cultures were spotted on LB agar plates containing either 1% glucose or 0.2% arabinose, and 200 ng·mL^−1^ AHTC, and the plates were incubated at 37 °C for 18 h.

### Immunodetection

Protein samples were analyzed by standard SDS/PAGE and western blot. Immunodetection was performed using primary anti‐FLAG mouse antibody (Clone M2; Merck, Darmstadt, Germany), and anti‐mouse alkaline phosphatase‐conjugated secondary antibody (Jackson ImmunoResearch, Cambridge, UK).

### *In vitro* synthesis of (p)ppApp or (p)ppGpp coupled with HPLC analysis

These experiments were performed as previously described [11, 18]. Briefly, the *in vitro* pppApp or pppGpp synthesis reaction was carried out in 10 mm Tris–HCl pH 8, 100 mm NaCl, and 15 mm MgCl_2_ containing 5 mm of nucleotide substrates (ATP alone or with GTP) and 1 μm of purified enzyme, and up to 50 μm for enzymes whose Syn motifs had been modified (see Ref. [11] for enzyme purification process). After incubation for 2 h at 37 °C, the reaction mixture was passed through a spin filter column and injected onto an HPLC system equipped with a strong anion exchange analytical column (SAX, Waters Spherisorb). Nucleotide separation was performed using an ionic strength gradient from solvent A (50 mm KH_2_PO_4_ pH 3.4) to solvent B (1 m KH_2_PO_4_ pH 3.4).

### Bacterial two‐hybrid

Plasmids allowing the production of proteins fused to the T18 or T25 domains of the Bordetella pertussis adenylate cyclase were cotransformed in *E. coli* BTH101. Bacteria were grown overnight in LB supplemented with 0.5 mm IPTG, and 2 μL were spotted on MacConkey agar medium containing 1% maltose.

## Results

### The predicted adenosine 3′‐pyrophosphokinase domains encoded by *M. haemolytica* and *P. multocida* prophages are toxic in *E. coli*

**Fig. 1:**
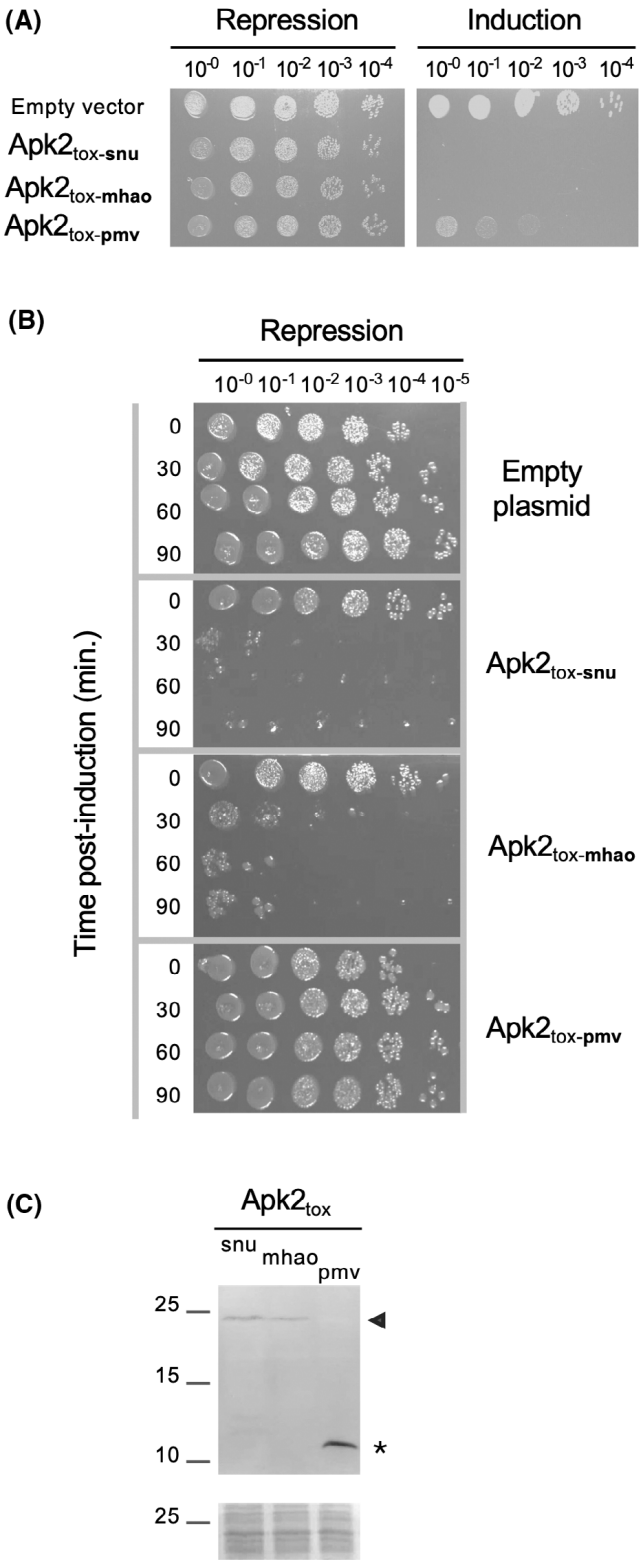
Toxicity of *Streptococcus pneumoniae*, *Mannheimia haemolytica*, and *Pasteurella multocida* Apk2_tox_ domains in *Escherichia coli*. (A) Toxicity assays—*E. coli* transformed with pBAD33 vectors encoding the toxic C‐ter domain of Apk2 from *S. pneumoniae*, *M. haemolytica*, or *P. multocida* (Apk2_tox‐snu_, Apk2_tox‐mhao_ or Apk2_tox‐pmv_, respectively) were grown to mid‐exponential phase, and serial dilutions were spotted on selective medium that repressed or induced *apk2*_*tox*_ expression. Data shown are representative of *n* ≥ 3 experiments. (B) Survival assays—*E. coli* transformed with pBAD33 vectors encoding Apk2_tox‐snu_, Apk2_tox‐mhao_, or Apk2_tox‐pmv_ were grown to mid‐exponential phase. After induction of the *apk2*_*tox*_ genes for 0–90 min as indicated, culture samples were harvested, washed, serially diluted, and spotted onto selective medium that repressed the expression of these genes. The experiment was performed twice. (C) Protein production—Genes encoding FLAG‐tagged catalytic‐null variants of Apk2_tox_ domains were induced in *E. coli* for 5 h. Proteins from the  corresponding cell extracts were separated by SDS/PAGE, and FLAG‐tagged Apk2_tox_ domains were immunodetected. Full‐length form and degradation products are indicated by a black arrowhead and the asterisk, respectively. Molecular weight markers are indicated on the left (in kDa). The panel on bottom shows a piece of membrane stained with Ponceau red as a control for protein loading. Data shown are representative of *n* = 3 experiments.

Investigation of the distribution of (p)ppApp synthetases revealed the existence of two families. Both families have a C‐terminal domain with 3′‐adenosine pyrophosphokinase activity but differ in their N‐terminal domain: PAAR for the Apk1 family and MuF for the Apk2 family. Eighty‐nine Apk2 homologous sequences were found and were distributed across multiple bacterial phyla such as Pseudomonadota, Bacteroidota, Bacillota, and Actinomycetota [12]. Two recent studies characterized the (p)ppApp synthetase activity of the C‐terminal domain of Apk2 (Apk2_tox_) encoded by prophages from *Bacteroides caccae* and from *S. pneumoniae* that belong to the Bacteroidota and Bacillota phyla, respectively [11, 12]. In this study, we extended the characterization to two new members encoded by prophages from *M. haemolytica* and *P. multocida* that belong to the Pseudomonadota phylum. To evaluate and compare the toxicity of the Apk2_tox_ domains, corresponding coding sequences of *S. pneumoniae*, *M. haemolytica*, and *P. multocida* (hereafter referred to as Apk2_tox‐snu_, Apk2_tox‐mhao_ and Apk2_tox‐pmv_, respectively) were cloned into the pBAD33 vector under the control of the P_BAD_ promoter and an attenuated (5G) ribosome binding site (RBS). These domains were then heterologously produced in *E. coli*. Serial dilutions of *E. coli* cultures transformed with the empty vector or with the pBAD33‐*apk2*_tox‐snu_, pBAD33‐*apk2*_tox‐mhao_, and pBAD33‐*apk2*_tox‐pmv_ constructs were plated on LB agar containing glucose or arabinose to repress or induce the P_BAD_ promoter, respectively (Fig. 1A). While the growth of bacteria transformed by the different plasmids was comparable under repression conditions, the production of Apk2_tox_ domains conferred growth inhibition. The Apk2_tox‐mhao_ domain displayed a high toxic activity, similar to that of the Apk2_tox‐snu_ domain, whereas the Apk2_tox‐pmv_ domain was less toxic in *E. coli*.

### Heterologous production of the *S. pneumoniae* and *M. haemolytica* Apk2_tox_ domains is bactericidal to *E. coli*

To test whether the Apk2_tox_ toxins have a bactericidal or bacteriostatic effect, a survival assay was performed. For this, samples of *E. coli* cultures producing the Apk2_tox_ domains were harvested at different induction times (0–90 min) and spotted on solid medium under repressive conditions. This allowed us to estimate the number of cells that have survived the induction period. Figure 1B shows that the activity of Apk2_tox‐snu_ and Apk2_tox‐mhao_ was bactericidal, since the bacterial growth observed on repressive solid media was reduced as the induction time for domain production increased. In contrast, Apk2_tox‐pmv_ did not kill *E. coli*, as bacterial growth was comparable to that of *E. coli* cells bearing the empty vector, even after up to 90 min of induction.

### Estimation of Apk2_tox_ domain levels in heterologous host *E. coli*

The previous experiment showed that transient production of Apk2_tox_ of *S. pneumoniae* or *M. haemolytica* in *E. coli* is bactericidal, whereas transient production of Apk2_tox_ of *P. multocida* is not, even though the experimental setup was the same: same promoter, same RBS, and synthetic DNA optimized for *E. coli*. These results echo those of another study in which it was shown that the production of Apk1_tox_ from *Pseudomonas aeruginosa* in the heterologous host *E. coli* had a bactericidal effect, whereas production of Apk2_tox_ from *B. caccae* had a bacteriostatic effect [12]. Taken together, these results raised the question of whether Apk_tox_ domains have activities with different efficiencies or whether a production/stability issue occurred despite identical production conditions.

To address this question, the amount of Apk2_tox_ domains in *E. coli* was examined by western blot. Protein extracts were therefore prepared from *E. coli* strains producing FLAG‐tagged Apk2_tox_ domains (which displayed toxicity identical to untagged versions, Fig. S1) for 5 h. To avoid toxicity during the experiments, the key aspartate residues of the catalytic site of Apk2_tox_ domains were substituted by glycine. The proteins were then separated by SDS/PAGE, and the Apk2_tox_ FLAG‐tagged domains were immunodetected with the anti‐FLAG antibody. The Apk2_tox‐snu_ and Apk2_tox‐mhao_ tagged domains were detected at the expected size (~ 23 kDa) (Fig. 1C). According to the different replicates, Apk2_tox‐snu_ and Apk2_tox‐mhao_ were produced in comparable amounts or slightly less for the second one, an observation that is consistent with the results of the survival assays, in which the Apk2_tox‐mhao_ was reproducibly slightly less toxic than Apk2_tox‐snu_ (Fig. 1B). The Apk2_tox‐pmv_ tagged domain was not detected at the expected size, but a degradation fragment was detected at ~ 11 kDa, indicating that Apk2_tox‐pmv_ was cleaved and explaining the diminished toxicity observed for Apk2_tox‐pmv_ in *E. coli*.

**Fig. 2:**
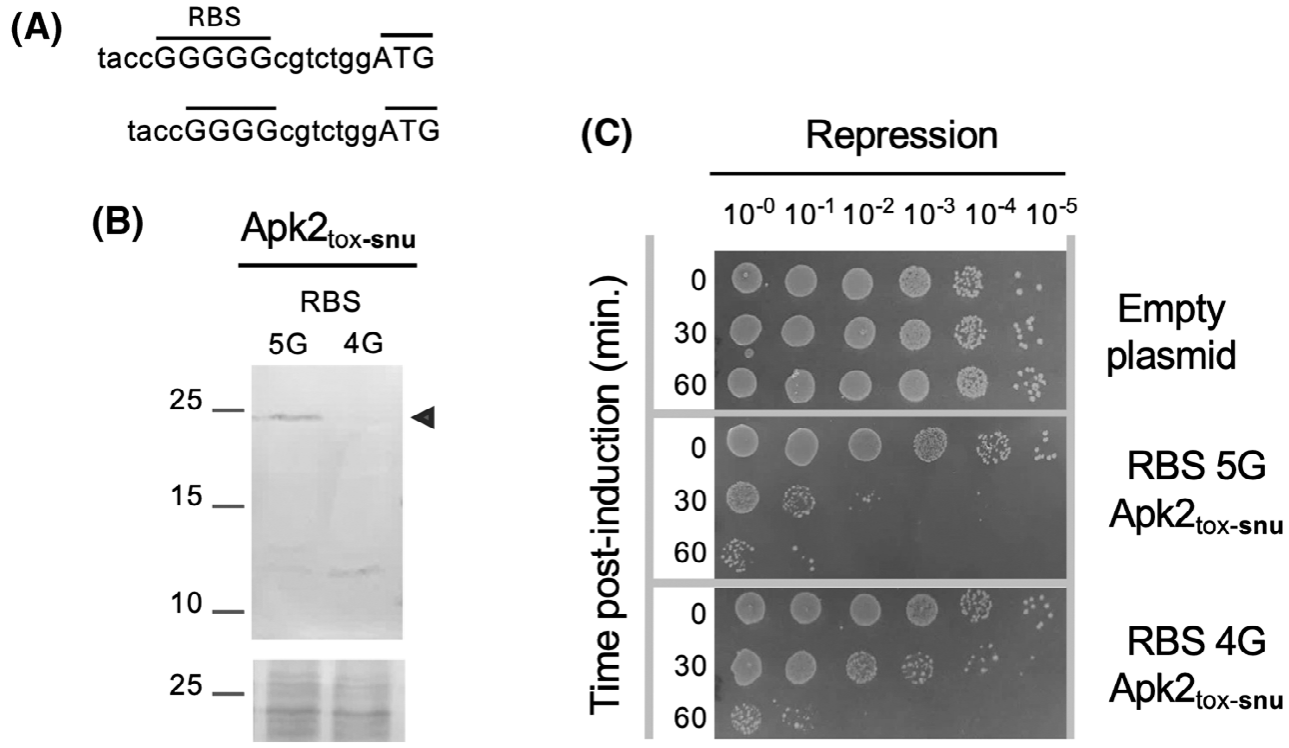
Correlation between Apk2_tox_ quantity and toxicity. (A) Sequences of the Apk2_tox‐snu_ 5G and 4G suboptimal ribosome binding site (RBS) variants. (B) Protein production—The FLAG‐tagged catalytic‐null variant of Apk2_tox‐snu_ was produced in *Escherichia coli* for 5 h using a 5G or 4G suboptimal RBS. Proteins from the corresponding cell extracts were separated by SDS/PAGE, and the FLAG‐tagged Apk2_tox‐snu_ domain was immunodetected. The full‐length form is indicated by a black arrowhead (expected mass ~ 23 kDa, molecular weight marker on the left). The panel on the bottom shows a piece of membrane stained with Ponceau red as a control for protein loading. Data shown are representative of *n* = 3 experiments. (C) Survival assays—*E. coli* transformed with pBAD33 vectors encoding the Apk2_tox‐snu_ gene cloned downstream of a suboptimal 5G or 4G RBS were grown to mid‐exponential phase. After induction of the Apk2_tox‐snu_ domain for 0–60 min as indicated, culture samples were harvested, washed, serially diluted, and spotted onto selective medium that repressed the expression of *apk2*_*tox‐snu*_. The experiment was performed twice.

To unambiguously correlate protein level and toxicity without working with different domains, Apk2_tox‐snu_ production and toxicity were compared in the heterologous host *E. coli* using the initial attenuated 5G RBS or a further suboptimal (4G) RBS (Fig. 2A). Immunodetection showed a significant difference in the production of Apk2_tox‐snu_ under the control of the 4G RBS compared to the 5G (Fig. 2B). In our western blot experiments, we observed that an induction time of 5 h to produce Apk2_tox‐snu_ allowed the accumulation of the full‐length domain, whereas a shorter induction time led to the detection of majoritarian degradation products. We suggest that a degradation system must be saturated to observe the full‐length form and that using a lower RBS (4G) reduces production, preventing saturation of the degradation system and making the full‐length form more difficult to detect compared to the degradation products. This decrease correlated with an attenuated bactericidal effect (Fig. 2C), demonstrating that differences in toxicity can be explained by differences in heterologous production or stability.

### *In vitro* synthesis of pppApp by Apk2_tox_ domains

**Fig. 3:**
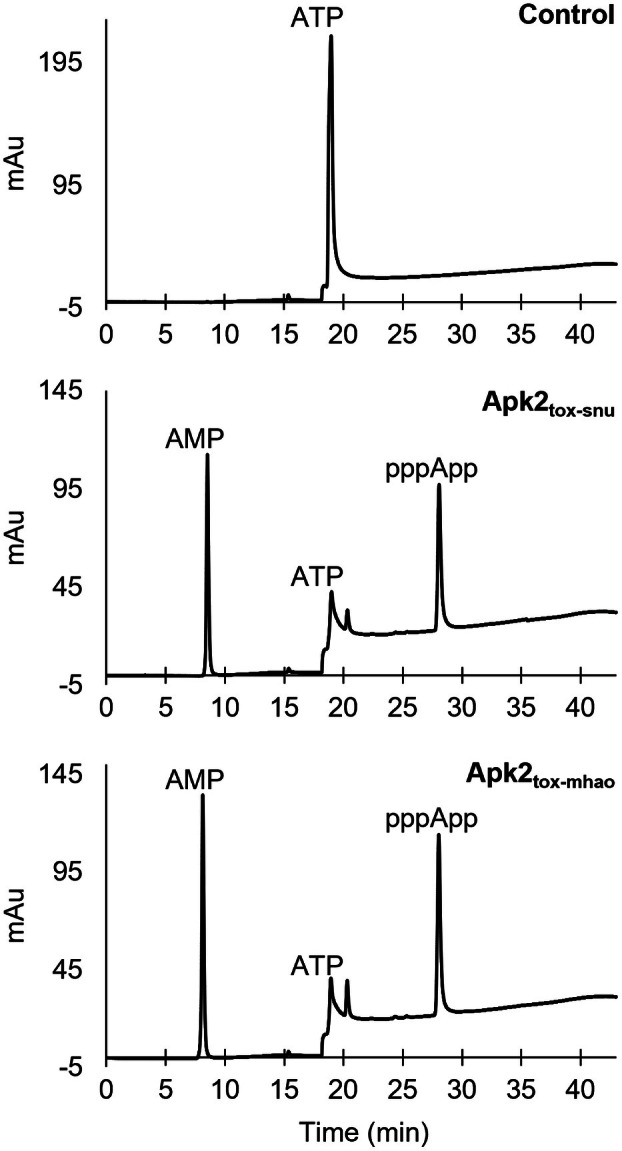
SAX‐HPLC analysis of nucleotides after *in vitro* reactions with Apk2_tox_ domains. Strong anion exchange high‐performance liquid chromatography (SAX‐HPLC) chromatograms of nucleotide products of *in vitro* synthesis reactions, with ATP as substrate and the indicated purified Apk2_tox_ domain. The control chromatogram (top panel) corresponds to a reaction that did not contain any enzyme. The peaks corresponding to AMP and ATP were determined with control experiments using commercial nucleotides. The experiment was performed twice.

To confirm the predicted adenosine 3′‐pyrophosphokinase activity of the Apk2_tox_ domains, *in vitro* pppApp synthesis reactions were performed. Purified Apk2_tox‐mhao_ domain was incubated with ATP for 2 h at 37 °C, and the products were analyzed by strong anion exchange high‐performance liquid chromatography (SAX‐HPLC). Apk2_tox‐snu_, for which the adenosine 3′‐pyrophosphokinase activity was previously characterized [11], was used as a positive control. The instability of Apk2_tox‐pmv_ prevented its production and purification. The chromatograms obtained after SAX‐HPLC analysis showed a single peak corresponding to ATP substrate in the control reaction without enzyme, a peak that decreased in the reactions with the Apk2_tox‐snu_ and Apk2_tox‐mhao_ domains, reflecting ATP consumption (Fig. 3). Two additional peaks appeared in the same samples, corresponding to AMP and pppApp, both products of the transfer of a pyrophosphate group from one ATP molecule to another. When similar experiments were performed with both ATP and GTP as substrates, no pppGpp was detected (Fig. S2, left panels), confirming that the Apk2_tox‐mhao_ domain, like the previously characterized Apk2_tox‐snu_ domain, specifically synthesizes pppApp.

### Conversion of the syn2‐3‐4 motifs of Apk2_tox_ to syn motifs of guanosine 3′‐pyrophosphokinase does not change nucleotide specificity

Based on a multiple protein sequence alignment comparing guanosine 3′‐pyrophosphokinase RSH members with predicted or proven adenosine 3′‐pyrophosphokinases, a previous study identified specific signatures at conserved syn sites that differ from one family to the other [11]. We thus investigated whether it was possible to convert a pppApp synthetase into a pppGpp synthetase by introducing substitutions at the syn motifs of the Apk2_tox‐snu_ domain. The Apk2_tox‐snu_ syn2, syn3, and syn4 motifs were substituted to correspond to consensus sequences associated with RSH. The GxN residues were converted to SxH at the syn3 site, which has been described to recognize guanosine in RSH [16], while the RYT and YH residues of the syn2 and syn4 motifs, respectively, were converted to AVR and IR. The Apk2_tox‐snu_ domain variants were purified and used in *in vitro* synthesis reactions with SAX‐HPLC analysis of the products. Introduction of these substitutions resulted in progressive inactivation of the Apk2_tox‐snu_ adenosine 3′‐pyrophosphokinase activity but did not lead to pppGpp production (Fig. S2, right panels). The failure to convert the Apk2_tox‐snu_ (p)ppApp synthetase to a (p)ppGpp synthetase by substituting conserved amino acids in the syn motifs suggests either that additional amino acids are key to control the specificity of the substrate or of the reaction, or that the environment provided by the protein backbone is not suitable for producing a functional enzyme after these substitutions.

### Interchangeability of the Apk2_tox_ immunity proteins

The *apk2* genes are followed by two genes, *iapK* (immunity of apk) and *aph1* (adenosine 3′‐pyrophosphohydrolase), which encode immunity proteins. IapK was shown to neutralize Apk2_tox_ by occluding the active site of the toxin, whereas Aph1 cleaves the pyrophosphate group of pppApp, hence regenerating the ATP pool [11, 12]. To assess the interchangeability of the immunity proteins whose genes are genetically linked to the *S. pneumoniae*, *M. haemolytica*, and *P. multocida muf* genes, we performed neutralization assays. For this, *E. coli* cells were cotransformed with a plasmid allowing the production of one of the Apk2_tox_ domains (snu, mhao, and pmv) and a plasmid allowing the production of one of the six immunity proteins. Cultures of the cotransformants were then serially diluted and spotted on LB agar supplemented with l‐arabinose and AHTC to induce the expression of the cloned toxin and immunity genes. The results demonstrated that, as expected, a nonspecific cross‐immunity was provided by the different Aph1 proteins, which hydrolyze pppApp, the product of the reaction catalyzed by the different Apk2_tox_ domains (Table 1 and Fig. S3). However, in our experimental setup, neutralization was only partial, except in the case of Apk2_tox‐pmv_, whose toxicity is low. By contrast, we observed an orthologous relationship for the IapK immunity proteins: Each Apk2_tox_ domain toxicity was neutralized by its cognate IapK immunity (Table 1 and Fig. S3). However, IapK_pmv_ was able to fully protect cells from Apk2_tox‐snu_ toxicity and we noticed a decreasing gradient of versatility: IapK_pmv_ > IapK_snu_ > IapK_mhao_.

**Table 1:** Toxicity neutralization assays. The evaluation of the toxicity level upon heterologous production in *Escherichia coli* is ranked strong (++) or intermediate (+) (Fig. 1A). The rescue profile based on several experiments is summarized in this table. The evaluation of the neutralization is ranked total (++), intermediate (+, small colonies or visible at lower dilutions than with total neutralization), and no neutralization (−) (Fig. S3).

Toxicity			Neutralization						
			Empty plasmid	IapK			Aph1		
				snu	mhao	pmv	snu	mhao	pmv
Apk2_tox_	snu	++	−	++	−	++/+	+	+	+
	mhao	++	−	−	++	+/−	+	+	+
	pmv	+	−	+/−	−/+	++	++	++	++

**Fig. 4:**
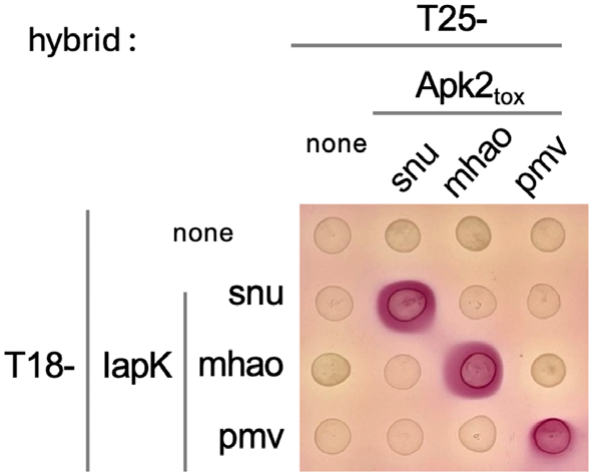
Apk2_tox_ domain and IapK immunity protein–protein interactions. Bacterial two‐hybrid analysis—Drops of cultures of *Escherichia coli* BTH101 reporter cells producing the FLAG‐tagged catalytic‐null variant of indicated Apk2_tox_ domains and IapK immunity proteins fused to the T25 and T18 domains of the *Bordetella pertussis* adenylate cyclase, respectively, were spotted on McConkey agar medium. The red color of the colony reports the reconstitution of the adenylate cyclase due to the interaction between the tested proteins. Data shown are representative of *n* = 4 experiments.

To further understand the specificity of cross‐neutralization between Apk2 toxins and IapK immunities, protein–protein interactions were assessed by bacterial two‐hybrid. Figure 4 shows that a strict orthologous relationship exists between cognate pairs. Surprisingly, while the toxicity neutralization assay indicated that IapK_pmv_ protects from Apk2_tox‐snu_ toxicity, these two proteins do not interact in the bacterial two‐hybrid assay. A possible interpretation could be that in the neutralization experimental setup (i.e., the toxin/immunity ratio is in favor of the immunity protein), an immunity protein with low affinity for a toxin could compensate by its higher production level. But, an interaction that would be too labile and/or dynamic may not be captured by the bacterial two‐hybrid method, as we have previously observed [19].

## Discussion

Three previous studies characterized polymorphic toxins with adenosine 3′‐pyrophosphokinase (Apk) activity from the Apk1 family, which is associated with the PAAR domain of the Type VI secretion system (T6SS) [13] and from the Apk2 family, which is associated with MuF phage proteins [11, 12]. This study extends the characterization of this new family of enzymes to include two new members of the Apk2 family encoded by bacteriophages from the Gram‐negative bacteria *M. haemolytica* and *P. multocida*. Overall, our results on these Apk domains indicate that (a) heterologous production of these domains in *E. coli* is toxic, (b) protein levels correlate with toxicity, with a potential bactericidal effect, (c) these enzymes catalyze the exclusive synthesis of (p)ppApp, (d) substitution of the conserved syn2, 3, and 4 motifs is not sufficient to change the specificity of the reaction toward (p)ppGpp synthesis, and (e) IapK immunity proteins are orthologous to their cognate Apk2_tox_ domains with more or less pronounced stringency.

Due to its instability, we were not able to demonstrate the adenosine 3′‐pyrophosphokinase activity of the Apk2_tox_ domain of *P. multocida*. However, this potential activity is also supported by the fact that the *aph1* gene, which is genetically linked to it, possesses the typical signatures of (p)ppApp hydrolases and all the conserved active site residues that determine the specificity for (p)ppApp [12].

Aph1 immunity proteins, which are enzymes hydrolyzing the (p)ppApp product, share between 43% and 57% identity (Table 2). On the other hand, the percentage of identity of IapK immunity proteins drops to 35–38% (Table 2) and probably reflects the adaptation of IapK to their cognate toxin, given their mode of action by protein–protein interaction and occlusion of the active site. Our bacterial two‐hybrid results highlight this orthogonality. However, our experimental setup in the toxicity neutralization assays revealed a slight versatility with IapK_pmv_, which provides protection against Apk2_tox‐snu_. Does this observation reflect an evolutionary history? *M. haemolytica* and *P. multocida* being quite distant in the bacterial phylogenetic tree from *S. pneumoniae*, one may hypothesize that prophage elements moved from one strain to the other by horizontal gene transfer. While it is difficult to conceive that the prophage elements were acquired by transduction because of the specificity of phages for bacterial species, *Streptococcus* and *Pasteurella* strains are naturally competent and are well known for their capacity to acquire foreign DNA by transformation [20, 21].

**Table 2:** Percentage of identity between Apk2_tox_ domains and IapK and Aph1 immunities based on Omega Clustal protein sequence alignments.

	Apk2_tox_			IapK			Aph1		
	snu	mhao	pmv	snu	mhao	pmv	snu	mhao	pmv
snu	100	44	46	100	35	38	100	45.2	42.7
mhao	44	100	45.5	35	100	36.6	45.2	100	57
pmv	46	45.5	100	38	36.6	100	42.7	57	100

It is noteworthy that these bacterial species occupy the upper respiratory tract. However, *Mannheimia* and *Pasteurella* contribute to bovine respiratory diseases [20] while *S. pneumoniae* is an opportunistic pathogen of the upper respiratory tract responsible for human infections [21]. Human respiratory infections caused by *Pasteurella* are relatively rare, but some cases were reported in patients with chronic lung diseases [20] and bacteria of the genus *Streptococcus* are present in the bacterial microbiota of the bovine respiratory tract [22]. It would be interesting to decipher the dynamic exchange of genetic material, and notably of prophage elements, between bacterial strains in their natural habitat and to understand the physiological role of these prophages and of MuF polymorphic toxins.

## Conclusions

In conclusion, this study extends our understanding of polymorphic toxins with adenosine 3′‐pyrophosphokinase (Apk) activity by analyzing two new members of the Apk2 family. Their integrity/stability must be taken into account when comparing their activity/toxicity in the heterologous host *E. coli*. Two types of immunity proteins can counteract their toxicity, either by protein–protein interaction with the toxin or by hydrolysing the product and hence restoring the ATP pool. Although very similar to RSH proteins, the conversion of their (p)ppApp synthetase activity to (p)ppGpp synthetase is not straightforward and, if possible, would require further analysis. The physiological role of these polymorphic toxins remains to be established.

## Conflict of interest

The authors declare no conflict of interest.

## Peer review

The peer review history for this article is available at https://www.webofscience.com/api/gateway/wos/peer‐review/10.1002/2211‐5463.70038.

## Author contributions

EMP, JB, AK, CMP, ACT and JPV acquired and interpreted the data. JPV conceived the project. EMP and JPV wrote the paper. JB and EC edited the paper.

## Supporting information


**Fig. S1.** Toxicity of *S. pneumoniae*, *M. haemolytica* and *P. multocida* Apk2tox and Apk2tox FLAG‐tagged domains in *E. coli*.
**Fig. S2.** Strong anion exchange HPLC analysis of nucleotides after *in vitro* reactions with Apk2tox domains.
**Fig. S3.** Toxicity neutralization assays and evaluation of cross‐immunity.

**Table S1.** Bacterial strains.
**Table S2.** Plasmids.
**Table S3.** Primers.

## Data Availability

Supporting data are contained in the manuscript in the Supporting Information section.
