# Supplementary material for: Comparative study of adenosine 3′‐pyrophosphokinase domains of MuF polymorphic toxins
Source: FEBS Open Bio. 2025 Apr 15;15(7):1103–12. doi: 10.1002/2211-5463.70038 (PMC12226406; doi:10.1002/2211-5463.70038)
Supplement: Supplementary file 1 — Fig. S1. Toxicity of S. pneumoniae, M. haemolytica and P. multocida Apk2tox and Apk2tox FLAG‐tagged domains in E. coli. Fig. S2. Strong anion exchange HPLC analysis of nucleotides after in vitro reactions with Apk2tox domains. Fig. S3. Toxicity neutralization assays and evaluation of cross‐immunity. [file FEB4-15-1103-s001.pdf]

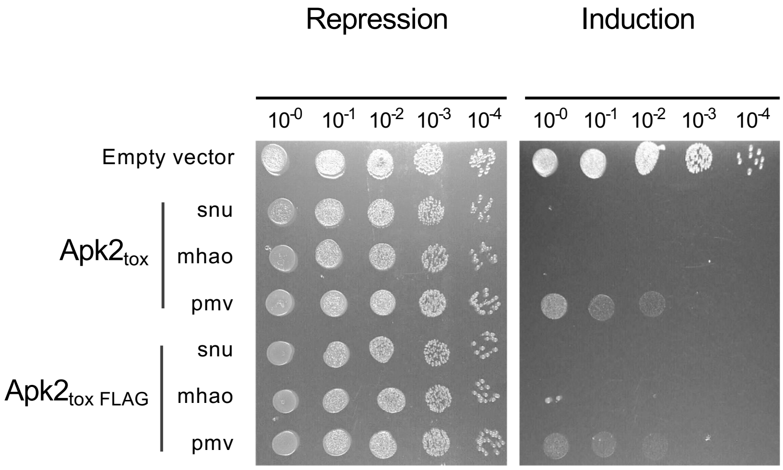

Figure SI 1 - **Toxicity of *S. pneumoniae*, *M. haemolytica* and *P. multocida* Apk2$_{tox}$ and Apk2$_{tox}$ FLAG-tagged domains in *E. coli***

E. coli transformed with pBAD33 vectors encoding Apk2$_{tox\text{-}snu}$, Apk2$_{tox\text{-}mhao}$ or Apk2$_{tox\text{-}pmv}$ or their FLAG-tagged versions were grown to mid-exponential phase and serial dilutions were spotted on selective medium that repressed or induced *apk2$_{tox}$* expression. The experiment was performed twice.

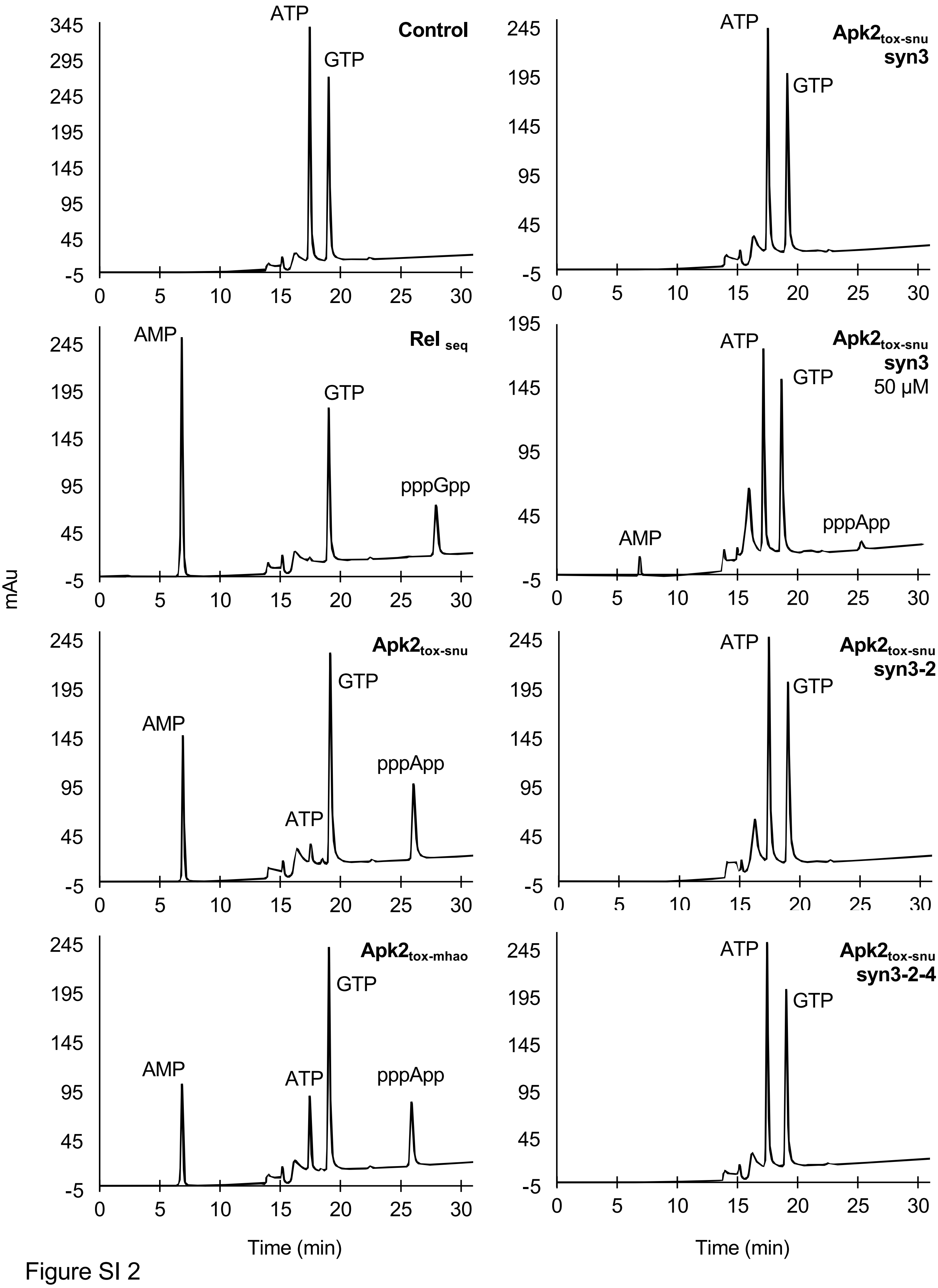
mAu
Time (min)
Control
ATP
GTP
Rel seq
AMP
GTP
pppGpp
Apk2tox-snu
AMP
ATP
GTP
pppApp
Apk2tox-mhao
AMP
ATP
GTP
pppApp
Apk2tox-snu syn3
ATP
GTP
Apk2tox-snu syn3 50 µM
AMP
ATP
GTP
pppApp
Apk2tox-snu syn3-2
ATP
GTP
Apk2tox-snu syn3-2-4
ATP
GTP

Figure SI 2

Figure SI 2– **Strong anion exchange HPLC analysis of nucleotides after *in vitro* reactions with Apk2$_{tox}$ domains**

Left panels- *In vitro* synthesis reactions were performed with the indicated purified domain and with ATP and GTP as substrates. Rel$_{seq}$ is the catalytic N-terminal fragment (residues 1 to 385) of the bifunctional RelA/SpoT homolog from *Streptococcus dysgalactiae subsp. Equisimilis*. The control chromatogram corresponds to a reaction that did not contain any enzyme. Right panels- *In vitro* synthesis reactions were performed with the Apk2$_{tox\text{-}snu}$ purified domains that had amino acid substitutions in the synthesis motifs (syn) as specified in the main text. ATP and GTP were provided as substrates. As no activity was detected for Apk2$_{tox\text{-}snu}$ with substitutions in the syn motifs when used at 1 µM like in the other assays, *in vitro* reactions were also performed with 50 µM of these variants. Apk2$_{tox\text{-}snu}$ with substitutions in the syn3 motif was the only one for which a weak residual 3'-adenosine pyrophosphokinase activity was detected in this condition. The peaks corresponding to regular nucleotides or pppGpp were determined with control experiments using commercial nucleotides.

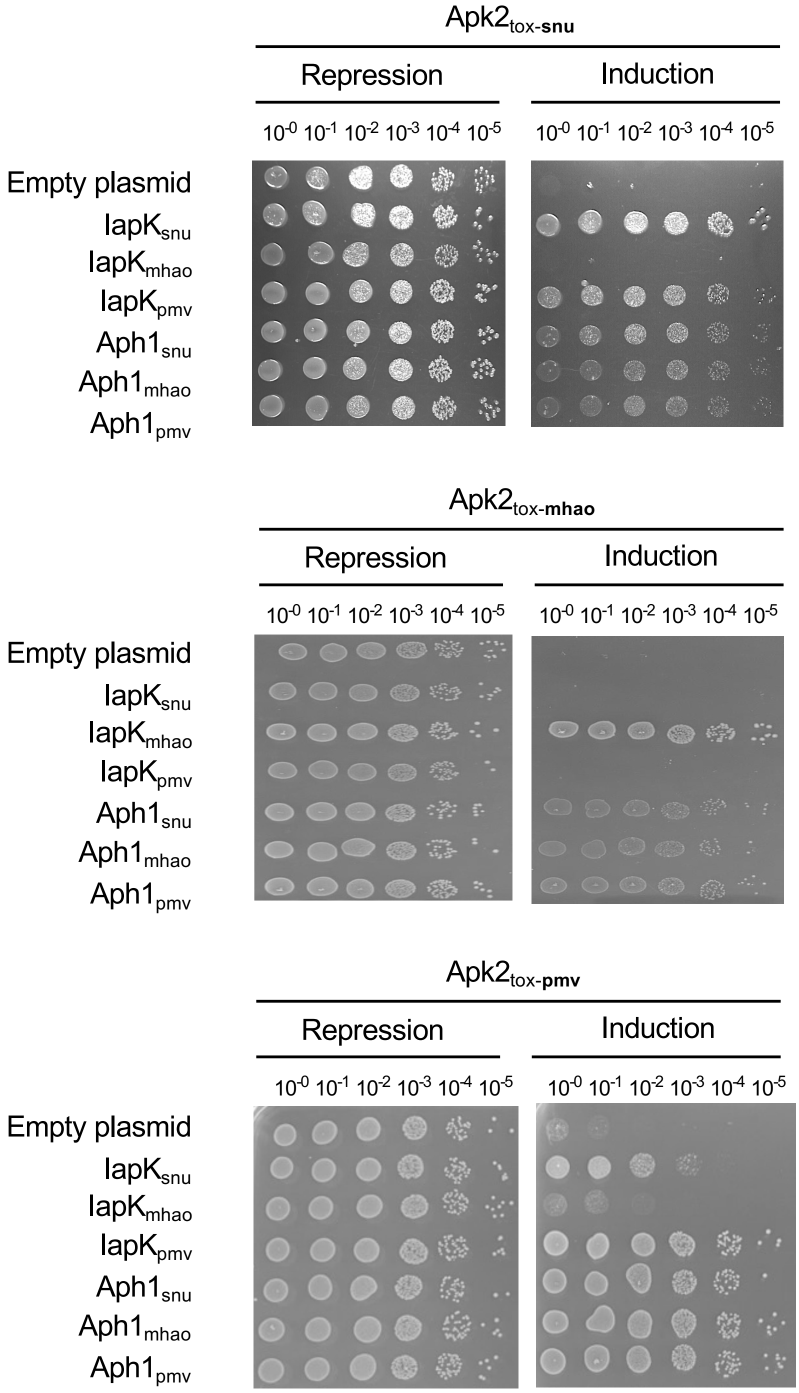

Figure SI 3- **Toxicity neutralization assays and evaluation of cross-immunity**

E. coli MG1655 cells were co-transformed with two plasmids and grown to mid-exponential phase before to be serially diluted and plated on LB agar plates containing anhydrotetracycline to induce the expression of the indicated (snu, mhao, pmv) *iapK* or *aph1* immunity genes from the first plasmid, and glucose or arabinose to repress or induce the expression of the indicated $apk2_{tox}$ from the second plasmid. Data shown are representative of n ≥3 experiments.
