# Supplementary material for: Comparative study of adenosine 3′‐pyrophosphokinase domains of MuF polymorphic toxins
Source: FEBS Open Bio. 2025 Apr 15;15(7):1103–12. doi: 10.1002/2211-5463.70038 (PMC12226406; doi:10.1002/2211-5463.70038)
Supplement: Supplementary file 2 — Table S1. Bacterial strains. Table S2. Plasmids. Table S3. Primers. [file FEB4-15-1103-s002.docx]

Table SI 1- **Bacterial strains**

| **Lab code** | **Name** | **Genotype** | **Reference** |
| --- | --- | --- | --- |
| EB3 | BTH101 | F^-^, *cya*-99, *araD*139, *galE*15, *galK*16, *rpsL*1 (Str r), *hsdR*2, *mcrA*1, *mcrB*1 | [1] |
| EB70 | DH5α | *fhuA*2, Δ(*argF*-*lacZ*)U169, *phoA*, *gln*V44, Φ80, Δ(*lacZ*)M15, *gyrA*96, *recA*1, *relA*1, *endA*1, *thi-*1, *hsdR*17 | Lab stock |
| EB72 | BL21(DE3) pLys | F^-^, *ompT*, *gal*, *dcm*, *lon*, *hsdSB*, (rB^-^ mB^-^), λ(DE3), pLysS(cm^R^) | Lab stock |
| EB944 | MG1655 | F^-^, λ^-^, *ilvG*^-^, *rfb*-50, *rph*-1 | Lab stock |

Table SI 2- **Plasmids**

| **Lab code** | **Description** | **Reference** |
| --- | --- | --- |
| Toxicity, survival and toxicity neutralization assays | | |
| pEB1017 | pBAD33 | [2] |
| pJV381 | pBAD33-apk2_tox-snu_ (amino acid residues 320 to 506 from the full-length Apk2_snu_ under the control of a RBS made of 5G) | [3] |
| pJV383 | pBAD33-apk2_tox-snu_ (as above but with a RBS made of 4G) |  |
| pJV390 | pBAD33-apk2_tox-snu_ D72G (D390G if full-length Apk2_snu_) (RBS made of 5G) | [3] |
| pJV391 | pBAD33-apk2_tox-snu_ D72G (D390G if full-length Apk2_snu_) (RBS made of 4G) |  |
| pJV421 | pBAD33-apk2_tox-mhao_ (amino acid residues 359 to 553 from the full-length Apk2_mhao_ under the control of a RBS made of 5G. PCR product w/ primers ebm2154/2155 at KpnI/SalI restriction sites) |  |
| pJV422 | pBAD33-apk2_tox-pmv_ (amino acid residues 346 to 533 from the full-length Apk2_pmv_ under the control of a RBS made of 5G. PCR product w/ primers ebm2152/2153 at KpnI/SalI restriction sites) |  |
| pJV445 | pBAD33-apk2_tox-snu_ 1xFlag (PCRi+SLIC, primers ebm2200/2201, template pJV381) |  |
| pJV446 | pBAD33-apk2_tox-mhao_ 1xFlag (PCRi+SLIC, primers ebm2202/2203, template pJV421) |  |
| pJV447 | pBAD33-apk2_tox-pmv_ 1xFlag (PCRi+SLIC, primers ebm2204/2205, template pJV422) |  |
| pJV457 | pBAD33-apk2_tox-snu_ D72G 1xFlag (RBS made of 5G) (mutagenesis, primers ebm2118/2119, template pJV445) |  |
| pJV458 | pBAD33-apk2_tox-snu_ D72G 1xFlag (RBS made of 4G) (PCRi+SLIC, primers ebm2200/2201, template pJV391) |  |
| pJV459 | pBAD33-apk2_tox-mhao_ D73G 1xFlag (D430G if full-length Apk2_mhao_)(mutagenesis, primers ebm2208/2209, template pJV446) |  |
| pJV460 | pBAD33-apk2_tox-pmv_ D72G 1xFlag (D416G if full-length Apk2_pmv_)(mutagenesis, primers ebm2210/2211, template pJV447) |  |
| pEB1242 | pASK-IBA37plus | IBA Lifesciences |
| pJV374 | pP_TET-_*iapK*_snu_ | [3] |
| pJV375 | pP_TET-_*aph1*_snu_ | [3] |
| pJV419 | pP_TET-_*iapK*_mhao_ (PCR product w/ primers ebm2166/2167 at EcoRI/XhoI restriction sites) |  |
| pJV420 | pP_TET-_*aph1*_mhao_ (PCR product w/ primers ebm2168/2169 at EcoRI/XhoI restriction sites) |  |
| pJV423 | pP_TET-_*iapK*_pmv_ (PCR product w/ primers ebm2160/2161 at EcoRI/XhoI restriction sites) |  |
| pJV424 | pP_TET-_*aph1*_pmv_ (PCR product w/ primers ebm2162/2163 at EcoRI/XhoI restriction sites) |  |
| Protein Production and Purification | | |
| pEB1520 | pETDuet-1 | Novagen |
| pJV403 | pETDuet-1-*apk2*_tox-snu_ *iapK*_snu_ | [3] |
| pJV435 | pETDuet-1-*apk2*_tox-mhao_ *iapK*_mhao_ (PCR products w/ primers ebm2181/2155 and ebm2179/2180 at EcoRI/SalI and NdeI/XhoI, respectively) |  |
| pJV439 | pETDuet-1-*apk2*_tox-snu_ syn3_mut_ *iapK*_snu_ (mutagenesis G436S N438H according to the coordinates of the full-length protein, primers ebm2134/2135) |  |
| pJV440 | pETDuet-1-*apk2*_tox-snu_ syn3-syn2_mut_ *iapK*_snu_ (further mutagenesis R393A Y394V T395R according to the coordinates of the full-length protein, primers ebm2170/2171) |  |
| pJV441 | pETDuet-1-*apk2*_tox-snu_ syn3-syn2-syn4_mut_ *iapK*_snu_ (further mutagenesis Y452I H453R according to the coordinates of the full-length protein, primers ebm2174/2175) |  |
| Bacterial Two-Hybrid | | |
| pEB354 | pKT25linker | [4] |
| pJV468 | pT25_Apk2_tox-snu_ D72G 1xFlag (PCR product w/ primers ebm2122/2212 at EcoRI/XhoI restriction sites) |  |
| pJV469 | pT25_Apk2_tox-mhao_ D73G 1xFlag (PCR product w/ primers ebm2207/2212 at EcoRI/XhoI restriction sites) |  |
| pJV470 | pT25_Apk2_tox-pmv_ D72G 1xFlag (PCR product w/ primers ebm2206/2212 at EcoRI/XhoI restriction sites) |  |
| pEB355 | pUT18Clinker | [4] |
| pJV397 | pT18_IapK_snu_ | [3] |
| pJV455 | pT18_IapK_mhao_ (PCR product w/ primers ebm2166/2167 at EcoRI/XhoI restriction sites) |  |
| pJV456 | pT18_IapK_pmv_ (PCR product w/ primers ebm2160/2161 at EcoRI/XhoI restriction sites) |  |

Table SI 3- **Primers**

| **Lab code** ebm | | **5’-3’ sequence** |
| --- | --- | --- |
| 2118 | AAAGCAGTTAGCAAAATTAACGgCGCTTTACGTTATACAACTATCTTT | |
| 2119 | AAAGATAGTTGTATAACGTAAAGCGcCGTTAATTTTGCTAACTGCTTT | |
| 2122 | gaagaattcatgGCGAAAGCTAAATTCTATAGTGAA | |
| 2134 | GATGGGCCATACAAAaGTGTAcACACGGTTGTCGAGAAAGATGG | |
| 2135 | CCATCTTTCTCGACAACCGTGTgTACACtTTTGTATGGCCCATC | |
| 2152 | ggtggtaccGGGGGcgtctggatgGTTGAGTCAGCTGTAAACGC | |
| 2153 | ctcctcgagCTAGTTGCGGATCTTACCAAT | |
| 2154 | ggtggtaccGGGGGcgtctggatgGTTGATAAAATGGACAGTGTGG | |
| 2155 | ctcctcgagTTACTTTTTTTCCTTGATCAGGT | |
| 2160 | gaagaattcATGGAATACTATTTAGTTTTTCCT | |
| 2161 | ctcctcgagTCACTTGTGTTTGTCAATCCAT | |
| 2162 | gaagaattcATGCACGGTTTGATCAGTATC | |
| 2163 | ctcctcgagTCAGGTTTGAAGAAATTGCTTC | |
| 2166 | ctcctcgagTCAGGTTTGAAGAAATTGCTTC | |
| 2167 | ctcctcgagTTAATGCACTTTAATAAAGCGGA | |
| 2168 | gaagaattcATGTGCCAAGCGGAGCAGT | |
| 2169 | ctcctcgagTCATGCCTGCAAGTATTCAAG | |
| 2170 | GTTAGCAAAATTAACGACGCTTTAgcTgtTcgtACTATCTTTGACTCTGATACTTTTAC | |
| 2171 | GTAAAAGTATCAGAGTCAAAGATAGTacgAacAgcTAAAGCGTCGTTAATTTTGCTAAC | |
| 2174 | GATGGTATCAATTTTGAAATGCAGatCCgCACACAGGAAAGCTTTGACCT | |
| 2175 | AGGTCAAAGCTTTCCTGTGTGcGGatCTGCATTTCAAAATTGATACCATC | |
| 2179 | catcatATGTACCAGTATTACCTGGCG | |
| 2180 | ctcctcgagATGCACTTTAATAAAGCGGATC | |
| 2181 | gaagaattcgATGGTTGATAAAATGGACAGTGTGG | |
| 2200 | tttatcatcgtcgtctttataatcTTTAACACGCTCAATGTTTTTTG | |
| 2201 | gattataaagacgacgatgataaaTGAcacgtgctcgacctgc | |
| 2202 | tttatcatcgtcgtctttataatcCTTTTTTTCCTTGATCAGGTCG | |
| 2203 | gattataaagacgacgatgataaaTAActcgacctgcaggcatg | |
| 2204 | tttatcatcgtcgtctttataatcGTTGCGGATCTTACCAATGTTC | |
| 2205 | gattataaagacgacgatgataaaTAGctcgacctgcaggcatg | |
| 2206 | gaagaattcATGGTTGAGTCAGCTGTAAACGC | |
| 2207 | gaagaattcATGGTTGATAAAATGGACAGTGTGG | |
| 2208 | CAAGCGATCGCCTCCATCCGCGgCGTGATTCGCTATACGGCGATTCTG | |
| 2209 | CAAGCGATCGCCTCCATCCGCGgCGTGATTCGCTATACGGCGATTCTG | |
| 2210 | GAATCACTTAGTAAAATCACCGgTATTGTGCGTTATACCACAATCTTC | |
| 2211 | GAAGATTGTGGTATAACGCACAATAcCGGTGATTTTACTAAGTGATTC | |
| 2212 | ctcctcgagTTAtttatcatcgtcgtctttataatc | |

References

1. Karimova G, Pidoux J, Ullmann A, Ladant D (1998) A bacterial two-hybrid system based on a reconstituted signal transduction pathway. *Proc Natl Acad Sci USA* 95, 5752–5756

2. Guzman LM, Belin D, Carson MJ, Beckwith J (1995) Tight regulation, modulation, and high-level expression by vectors containing the arabinose PBAD promoter. *J Bacteriol* 177, 4121–4130

3. Bartoli J, Tempier AC, Guzzi NL, Piras CM, Cascales E, Viala JP (2023) Characterization of a (p)ppApp Synthetase Belonging to a New Family of Polymorphic Toxin Associated with Temperate Phages. *Journal of Molecular Biology* 435:168282

4. Gully D, Bouveret E (2006) A protein network for phospholipid synthesis uncovered by a variant of the tandem affinity purification method in Escherichia coli. *Proteomics* 6, 282–293
